# Health Care Professionals' Confidence and Preferences for Diagnostic Assays for SARS-CoV-2: A Global Study

**DOI:** 10.3389/fpubh.2021.569315

**Published:** 2021-02-26

**Authors:** Adrian M. Shields, Hannah Brown, Neil Phillips, Mark T. Drayson, Anton A. Richter, Alex G. Richter

**Affiliations:** ^1^Clinical Immunology Service, Institute of Immunology and Immunotherapy, University of Birmingham, Birmingham, United Kingdom; ^2^M3 Global Research, London, United Kingdom

**Keywords:** SARS-CoV-2, COVID-19, global health, PCR testing, serological testing

## Abstract

**Background:** The COVID-19 pandemic has led to an urgent requirement for novel diagnostic tests that determine infection with SARS-CoV-2 and the development of an immune response against it. The perspective of end users on the characteristics and clinical use of these assays has not been previously considered.

**Methods:** We surveyed 17,186 health care professions (HCPs) in 29 countries to gauge opinion on the design, use, diagnostic impact and diagnostic accuracy of COVID-19 tests. Results were correlated with national statistics on the burden of disease and testing in individual countries.

**Results:** HCPs overwhelmingly recognized the importance of COVID-19 tests but 37.1% were unsure of the appropriate timing of investigations relative to disease symptoms. Confidence in the diagnostic accuracy of assays varied inversely with COVID-19-related mortality in individual countries but had no relationship with the total number of tests performed. There was global consensus that the most important impact of positive antigen and antibody testing was confidence in returning to work following recovery. Saliva was the preferred sampling fluid for COVID-19 diagnostic tests in all groups surveyed.

**Conclusions:** HCP input can ensure novel assays are fit for purpose in varied global health care settings, but HCPs may require support to effectively use novel diagnostics thus minimizing waste when supplies are limited.

## Introduction

As of January 2021, the coronavirus disease-19 (COVID-19) pandemic has resulted in over 93 million cases worldwide and over 2 million deaths (1). The pandemic has led to an urgent need for novel diagnostic tests that determine infection and immunity to SARS-CoV-2 at an individual and population level. Tests that directly confirm the presence of viral nucleic acids or proteins are used to shape national and international statistics on case burden and fatality rates (2), enable enrolment into clinical trials of therapeutics and supportive care and guide isolation policies for public health benefit.

While measurable, long-term correlates of immunological protection against SARS-CoV-2 remain unclear (3), cross-sectional sero-surveys have emerged as the favored tool in estimating prior exposure to the virus or response to vaccination and, by inference, the background herd immunity present at a population level. Early data suggests the presence of antibodies affords protection against viral re-infection (4). Ultimately, estimates of herd immunity will influence the stringency of local, national and international measures aimed at containing the spread of the virus. By extension, considerable controversy surrounds the concept of “immunological passports” that might facilitate individual relaxation of such measures (5).

The determinants of the quality and impact of diagnostic testing are multi-factorial and essential to understand in order to improve global health outcomes (6). At the level of the test itself, performance characteristics including the analytical sensitivity and specificity must be considered alongside the clinical pre-test probability. Tests detecting viral nucleic acids or proteins are time-dependent, displaying the greatest sensitivity and diagnostic utility shortly after symptom onset. Polymerase chain reaction (PCR) testing remains the gold standard method of case definition (2); the sensitivity of PCR tests depends on the type of body fluid analyzed (7) and time from symptom onset (8, 9). Rapid antigen tests have been deployed in asymptomatic screening programmes in an attempt to interrupt viral transmission (10, 11). These tests appear less sensitive that PCR but may be helpful in detecting individuals with relatively high-viral loads (PCR cycle thresholds <30) and guiding self-isolation policy (12, 13).

In contrast, antibody responses to viral infections take time to develop and antibody tests are only reliably sensitive two weeks after symptom onset (14, 15). Over 90% of PCR positive individuals mount a detectable antibody response following infection if a sufficiently sensitive assay is used for detection (16), but concern has been raised regarding the real-life performance of first-generation point lateral flow tests (17). Nevertheless, serological investigations may provide a helpful adjunct to acute molecular diagnostics, enhancing overall case detection, particularly in individuals testing negative by PCR (16, 18).

Arguably the most important factor determining the impact of COVID-19 laboratory diagnostics is understanding how health care professionals (HCPs), organizations, governments and individuals will use and interpret the results of such tests. There may be regional variation in these factors depending on access to diagnostics and disease burden.

Little attention has been paid to these concepts, or how diagnostics might be deployed in resource-limited settings where the capacity for testing is limited (19). To enable the design and implementation of future diagnostics, in collaboration with M3 Global Research, we sought the opinion of health care professionals from around the world.

## Methods

To understand health care professionals' (HCPs) opinions on COVID-19 diagnostic tests, we undertook an international study supported by the healthcare research company M3 Global Research. A short survey was devised (provided in full in the Supplementary Methods) and sent to approximately 100,000 participants. Participants were a random sample of HCPs from M3 Global Research and their global partners' existing databases. Participants were invited to participate in a 10 min online study by email. The survey was distributed in English and the local languages with the exception of India and the Nordic countries who received the English version only. There was no incentive for taking part and the study sponsors were not mentioned.

All respondents had previously opted in to receiving email invitations to online surveys. No follow-up or reminder emails were sent. The invitation clearly stated the aims and objectives of the study, the estimated length of interview, and that there was no incentive available for their participation, other than a copy of the report if requested. The research sponsor was not mentioned. All responses were pseudonymised so no respondents were identifiable, and no personal data was shared. The M3 Global Research privacy policy (https://www.m3globalresearch.com/research/privacy/) outlines how respondents' data will be processed, and participants can withdraw their consent to take part at any time during the process. Ethical approval for this study and written informed consent from the participants of the study were not required in accordance with local legislation and national guidelines.

Responses were collated by M3 Global Research and analyzed by the University of Birmingham. A total of 17,186 responses were received between 16th and 23rd April 2020. The overall response rate to the survey was similar to that of other M3 Global Research projects. The primary medical specialism of respondents is provided in Supplementary Table 1. At the time the survey was distributed, “antigen test” was the commonly used, albeit scientifically imprecise, term encompassing a variety of tests used to confirm acute SARS-CoV-2 infection including polymerase chain reaction and direct viral antigen detection.

Data on the global burden was extracted from public datasets collated by Our World in Data and available at *https://github.com/owid/covid-19-data/tree/master/public/data* and accessed on 23/4/2020. Confirmed cases and deaths were originally derived from the European Center for Disease Prevention and Control statistics. High Income Countries (HIC) and Low Middle Income Countries (LMIC) were defined by the Organization for Economic Co-operation and Development.

Data were analyzed using Graph Pad Prism 8.4.2 for macOS. Significant trends in ordinal survey data were analyzed using the Chi squared test for trend. World map figures were produced using Map Chart (*https://mapchart.net/world.html)*.

## Results

A total of 17,186 responses were received from 21 high income countries (HICs) and 8 low-middle income countries (LMICs) (Table 1). Respondents from high-income countries accounted for 82.8% of all respondents. 53.0% of respondents stated they worked in secondary care, 12.9% in primary care and 34.1% did not specify their specialism or place of work.

**Table 1:** Demographics of health care professionals participating in this study, the reported COVID-19 burden of disease and the total number of antigen tests performed in their respective countries.

	**Respondents (% total)**	**Primary care (%)**	**Secondary care (%)**	**Not specified (%)**	COVID-19 tests per 1000 population^*^	COVID-19 deaths per 10000 population^*^
Argentina	128	17.2	74.2	8.6	0.8	0.3
Australia	847	25.7	32.5	41.8	17.32	0.3
Austria	78	19.2	59.0	21.8	21.52	5.3
Belgium	130	13.8	52.3	33.8	14.27	51.6
Brazil	450	7.8	60.2	32.0	n.d.	1.3
Canada	366	21.3	44.3	34.4	15.05	4.9
China	609	1.3	58.8	39.9	n.d.	0.3
Colombia	180	43.3	52.2	4.4	1.22	0.4
Denmark	67	19.4	47.8	32.8	17.34	6.4
Finland	43	14.0	67.4	18.6	11.01	2.5
France	1001	12.8	34.5	52.7	7.05	31.6
Germany	575	10.4	50.8	38.8	20.94	5.9
India	797	21.8	56.2	22.0	0.32	0.0
Italy	1515	6.6	58.4	35.0	24.52	1.1
Japan	2046	10.6	71.5	17.9	0.98	0.1
Mexico	291	34.0	56.4	9.6	0.3	0.7
Netherlands	115	17.4	42.6	40.0	10.19	22.8
New Zealand	30	30.0	33.3	36.7	18.52	0.3
Norway	31	16.1	41.9	41.9	26.66	3.0
Poland	29	6.9	86.2	6.9	5.65	1.1
Russia	240	9.2	77.9	12.9	14.9	0.3
South Africa	167	9.0	24.0	67.1	2.16	0.1
South Korea	904	19.1	72.9	8.0	11.09	0.5
Spain	1035	15.1	52.2	32.8	20.02	45.8
Sweden	80	28.8	68.8	2.5	9.35	17.4
Switzerland	90	13.3	51.1	35.6	26.25	13.7
Turkey	334	21.0	68.3	10.8	8.51	2.7
United Kingdom	826	13.4	38.0	48.5	5.91	25.8
United States	4182	8.0	45.7	46.3	12.08	13.6
**Total**	**17186**	**12.9**	**53.0**	**34.1**		

* *Data on COVID-19 testing and deaths sourced from ourworldindata.org, accessed 24/4/2020. n.d, no data available*.

**Figure 1:**
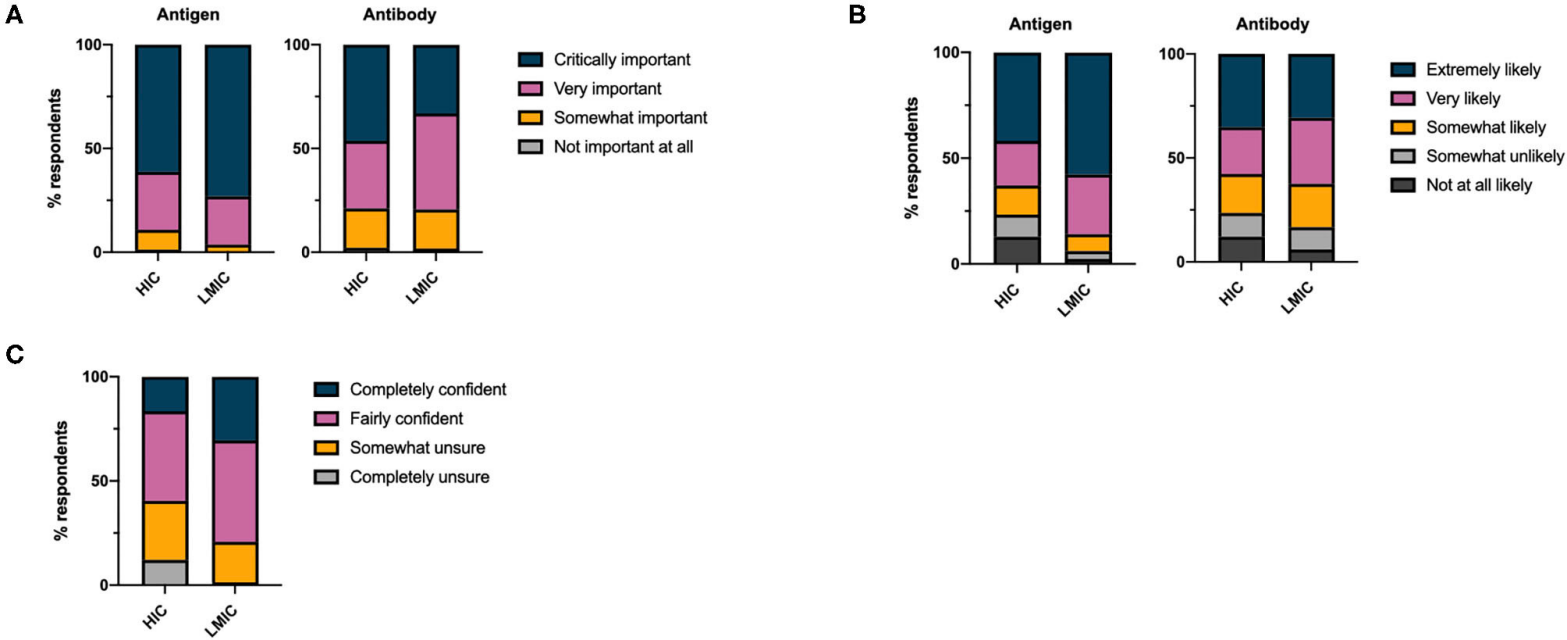
**(A)** Health care professionals' perceptions of the importance of antigen and antibody based diagnostics in controlling the COVID-19 pandemic. **(B)** Self-reported likelihood of health care professionals requesting antigen and antibody based diagnostic tests for COVID-19 disease. **(C)** Overall self-reported confidence of health care professionals in requesting COVID-19 diagnostic tests.

The overwhelming majority of respondents considered antigen tests critically important (63.3%) or very important (27.1%) in the control of the COVID-19 pandemic (Figure 1A). By comparison, fewer respondents considered antibody tests important for the control of the pandemic (critically important 44.0% or very important 34.9%, χ^2^ = 1513, df =3, *p* < 0.0001). LMICs attributed greater importance to antigen tests in the control of the pandemic than HICs (χ^2^ = 201.8, df = 3, *p* < 0.0001), while a greater percentage of respondents from HICs considered antibody tests of critical importance in pandemic control (χ^2^ = 228.9, df =3, *p* < 0.0001). Accordingly, the likelihood of respondents requesting antigen tests was significantly greater than that for an antibody tests overall (χ^2^ = 510.5, df =4, *p* < 0.0001, for trend); this effect was more marked when LMICs were compared to HICs (Figure 1B).

Despite respondents acknowledging the importance of antigen and antibody tests for COVID-19 and stating they would request these investigations, 37.1% of respondents were somewhat unsure or completely unsure regarding the appropriate timing of diagnostic testing with respect to the disease course. Uncertainty in the appropriate timing of testing was marginally greater amongst those working in secondary care (35.6%) than primary care (32.7%). Overall confidence in the timing of testing was significantly greater in LMICs compared to HICs (χ^2^ = 621.4, df =3, *p* < 0.0001) (Figure 1C). We found no linear relationship between the total number of tests per capita performed in different countries and the confidence of health care professionals in those countries in requesting tests. Considerably less testing has taken place in LMICs compared to HICs (Table 1).

**Figure 2:**
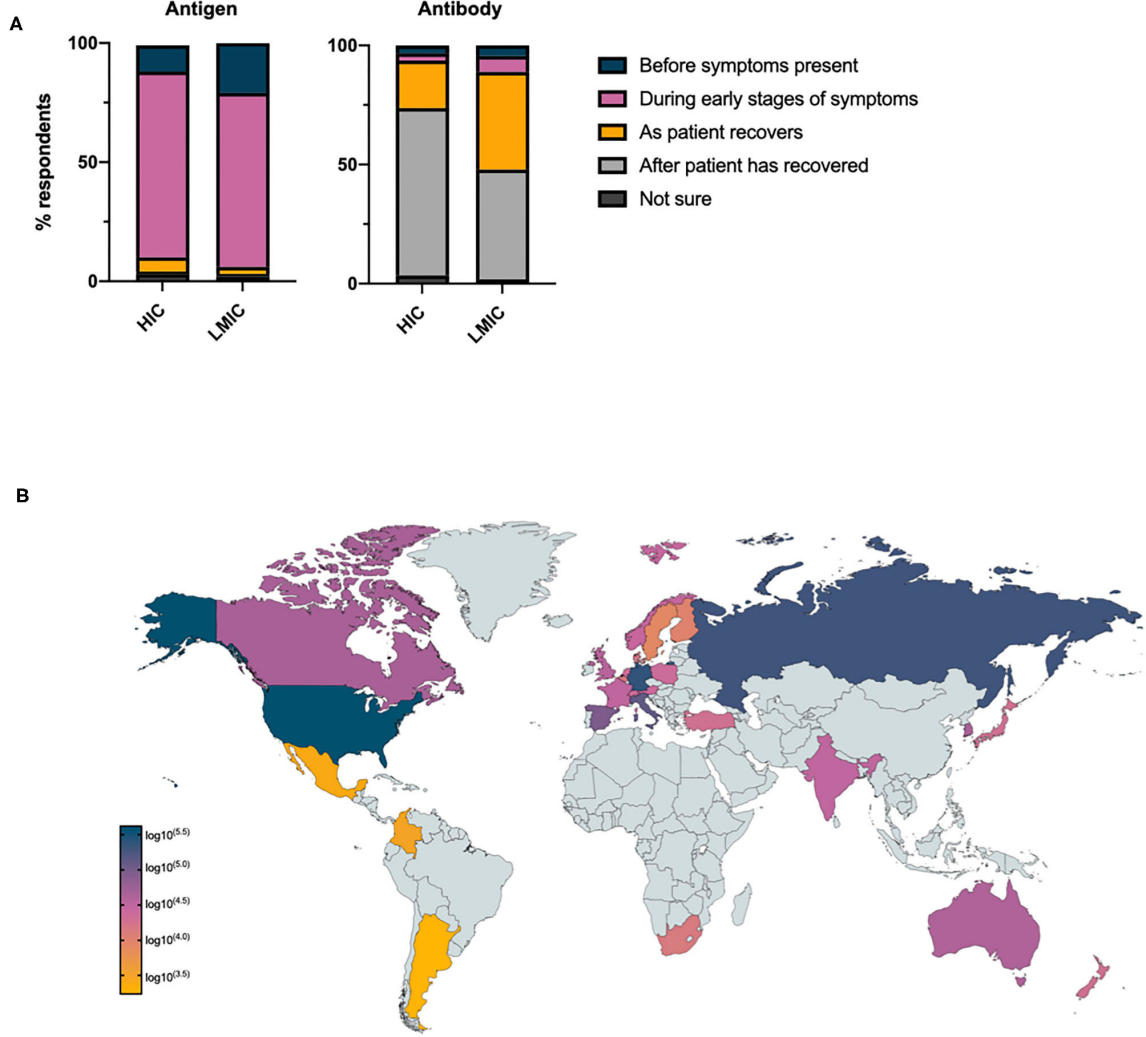
**(A)** Health care professionals' opinion on when antigen and antibody tests will demonstrate maximum accuracy in the assessment of COVID-19 relative to disease stage. **(B)** Total number of potentially sub-optimally requested COVID-19 tests requested per country based on national testing statistics.

Participants were asked at what stage of the disease they considered antigen and antibody tests to be most accurate (Figure 2A): despite a lack of self-reported confidence in the appropriate timing of COVID-19 diagnostics, 90.3% and 89.6% of respondents recognized antigen and antibody tests to be most accurate at appropriate stages of disease course (i.e., antigen tests before symptoms are present and during the early stages of symptoms, antibody tests as the patient recovers from their illness and after the patient has recovered from COVID-19). There was little difference between respondents in primary and secondary care with respect to the percentage of respondents who failed to recognize the appropriate timing of antigen tests (primary care 8.7% vs. secondary care 8.5% vs. no response 12.0%). There was no relationship between the self-reported confidence of respondents in the timing of COVID-19 diagnostics and knowledge of when antigen and antibody tests would be most accurate. A negative correlation was noted between the number of COVID-19 antigen tests requested per capita in individual countries, and the percentage of respondents who considered antigen tests to be most accurate outside of the pre-symptomatic or symptomatic phase of these disease (ρ = −0.4508, CI −0.7093–0.0854, *p* = 0.02). A similar relationship for antibody tests was not observed.

Given the global increase in antigen testing for COVID-19, and the uncertainty expressed by HCPs on when to request diagnostic investigations, we went on to consider the potential burden of sub-optimally requested COVID-19 antigen tests globally. Assuming, health care professionals who did not know when to optimally request an antigen test would still request such investigations and based on national testing figures, the potential global impact of this pre-analytical error may have exceeded 1,000,000 tests by April 2020 (Figure 2B).

**Figure 3:**
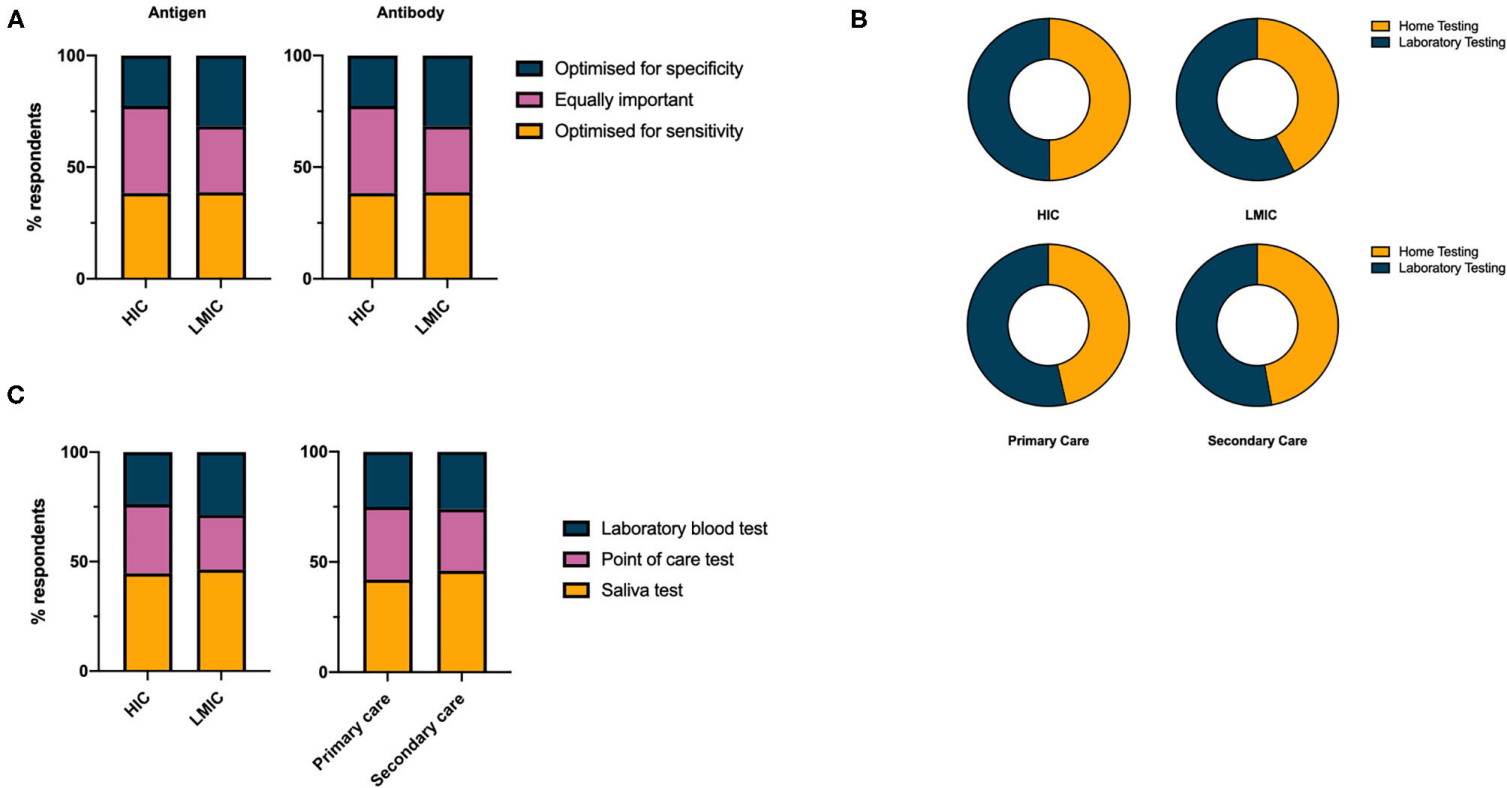
**(A)** Health care professionals' preferences for performance characteristics of COVID-19 antigen and antibody tests. **(B)** Health care professionals' preference for laboratory vs. home testing services for COVID-19 diagnostics. **(C)** Health care professionals' preference for sample used in COVID-19 diagnostic test.

There was no consensus from respondents on whether a COVID-19 assay should be optimized for sensitivity, specificity or whether both are equally important. For antigen tests, a small majority (41.3%), expressed the opinion both sensitivity and specificity were equally important, however opinion was more polarized in LMICs compared to HICs (Figure 3A). A slight overall preference for optimisation toward test sensitivity was noted for antibody tests (38.5%). A significantly greater proportion of respondents favored laboratory testing over home testing methods in LMICs compared to HICs (57.5% vs., 50.1%, χ^2^ = 54.42, df =1, *p* < 0.0001) however there is clearly demand for both modalities of test delivery (Figure 3B). Saliva was the preferred sampling fluid for COVID-19 testing in HICs and LMICs, and primary and secondary care (Figure 3C).

**Figure 4:**
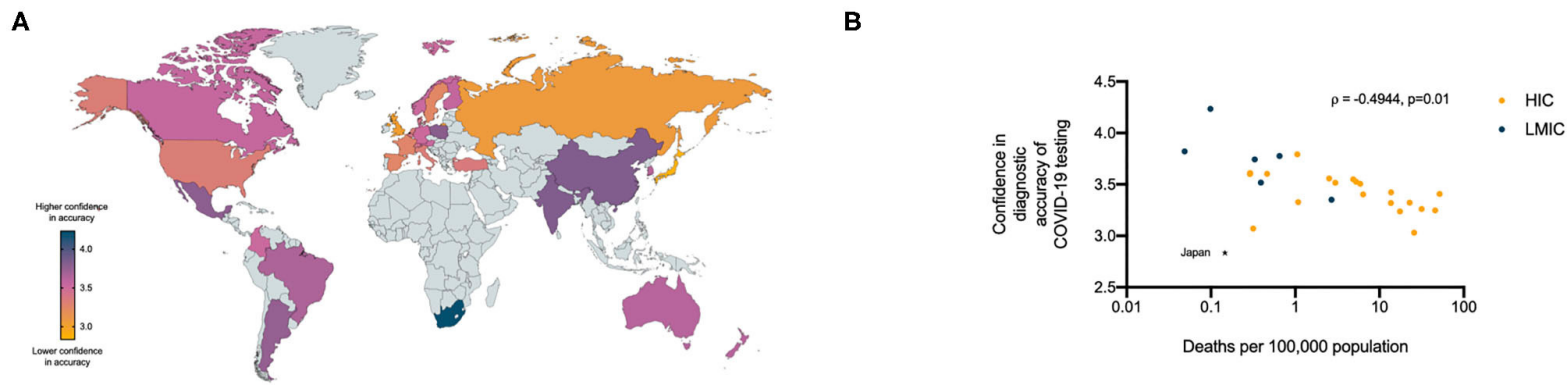
**(A)** Relative confidence of health care professionals in the diagnostic accuracy of currently available COVID- 19 tests per country (5 = high confidence, 1 = low confidence). **(B)** Relationship between health care professionals confidence in diagnostic accuracy of COVID-19 tests and the number of COVID-19 deaths per 100,000 population in respondents countries.

We noted marked international variability in the confidence of respondents in the accuracy of the COVID-19 diagnostic tests currently available to them (Figure 4A). With the exception of Japan, whose self-reported confidence in requesting diagnostic tests and confidence in the diagnostic accuracy of tests were both 2.5 standard deviation away from the mean of all HICs, a trend seen in other M3 surveys, we identified a negative correlation between confidence in the diagnostic accuracy of the tests and the number of deaths per capita documented in each country (ρ = −0.4944, CI −0.7400– −0.1324, *p* = 0.0102) (Figure 4B).

**Figure 5:**
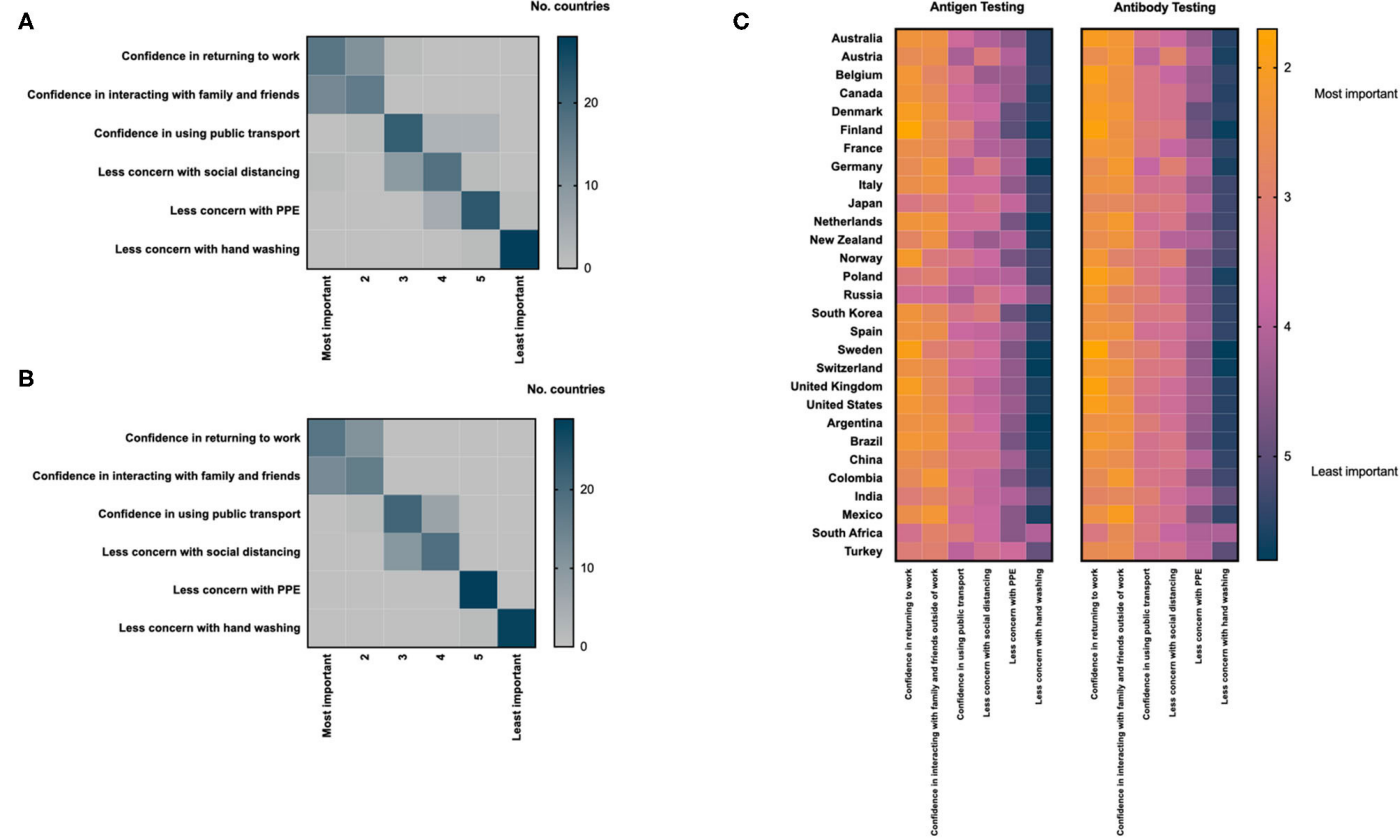
Health care professionals assessment of the impact of a positive antigen **(A)** or antibody test **(B)** on an individual level; heat maps are scaled by the total number of countries ranking each parameter by importance. **(C)** Differences in the assessment of the impact of positive COVID-19 antigen and antibody tests displayed by country; heat maps are scaled using the mean score for each parameter provided by respondents from individual countries.

There was global consensus on the impacts of positive antigen and antibody tests on individuals; confidence in returning to work was considered the most important impact of a positive antigen and antibody test, followed by confidence in interacting with family and friends, outside of work (Figures 5A,B). Reducing concern around personal protective equipment and hand-washing were considered least important. Minimal geographic variation was observed with respect to these patterns (Figure 5C and Supplementary Figures 1, 2). There was high concordance between the perceived impact of positive antigen and antibody tests in respondents from individual countries (Supplementary Figure 3).

## Discussion

Understanding the opinion and behaviors of HCPs who use COVID-19 diagnostics is critical in maximizing the utility and impact of each diagnostic test, particularly when access to diagnostic reagents and platforms is limited (20–22). Molecular testing is now an established measure in controlling the COVID-19 pandemic and their widespread use is reflected in the high likelihood of health care professionals requesting antigen testing seen in our data. Our survey was distributed in late April 2020, following the peak of the first wave of the pandemic in many countries. It is, therefore, concerning that as many as 9.7% of HCP approached did not understand the appropriate timing of antigen testing with respect to symptom onset.

Furthermore, we observed marked regional differences in health care professionals' confidence in the diagnostic accuracy of antigen tests. The causes of these differences are unclear: there was no relationship between the total number of tests ordered per capita and confidence in diagnostic accuracy of the tests. However, the negative correlation between confidence in the diagnostic accuracy of the tests and the number of deaths per capita documented in each country may provide insight into the known limitations of testing and be important in understanding health care professionals' behavior in test interpretation during the evolving pandemic. These observations merit further investigation, in particular, how the potential strengths and limitations of novel diagnostic tests for infectious diseases are communicated to health care professions, organizations and governments during rapidly evolving public health emergencies.

In resource limited settings, is critical that every test undertaken has maximum impact on the diagnostic or public health containment process. 37.1% of respondents in this survey were somewhat unsure or completely unsure as to what test should be undertaken in different stages of COVID-19 disease, yet 79.6 and 78.0% were likely to request antigen and antibody testing respectively. As the number of tests increased per capita, respondents were less likely to express the opinion that these tests were most accurate during the pre-symptomatic or symptomatic phase of the disease. This may reflect a lack of knowledge, or a diversification in what antigen tests are used for in these countries, for example screening. Given the rapid increase in COVID-19 testing, the impact on public health, health inequalities, and health economics of sub-optimally requested COVID-19 investigations also requires urgent attention.

Our data documents the varied perspectives of health care professionals on what types of test and what samples are most desirable with respect to COVID-19 diagnostic assays. Laboratory based testing was favored in primary care, secondary care and in LMICs and there was a preference for saliva as the sampling fluid of choice in respondents from all demographics. Saliva has shown superior performance to nasopharyngeal swabs for antigen testing for SARS-CoV-2 and is simple and non-invasive (23). Antibodies against bacteria and viruses can already be routinely detected in saliva with levels correlating to serum concentrations (24, 25). When translated into point of care lateral flow test (similar to a pregnancy test), the presence or absence of biomarkers of infection can be ascertained with no invasive sampling or further laboratory equipment, with implications for deployment in resource-limited settings. These findings should prompt assay development by academia and industry in collaboration with the end users and acknowledgment that a single test or platform might not be suitable for all applications.

In conclusion, the rapid global growth in the use of diagnostic assays for COVID-19 is an impressive achievement that has required the collaboration of multiple stakeholders including governments, industry and academia. Health care professionals, the end users of these novel assays, can provide invaluable insight into their diagnostic impact and help guide future assay development for different global settings.

## Data Availability Statement

The raw data supporting the conclusions of this article will be made available by the authors, without undue reservation.

## Ethics Statement

Ethical review and approval was not required for the study on human participants in accordance with the local legislation and institutional requirements. The patients/participants provided their written informed consent to participate in this study.

## Author Contributions

AAR and AGR conceived the study. HB, NP, and AAR collected data for the study. All authors critically appraised the data. AS wrote and corrected the manuscript following critical appraisal from all authors.

## Conflict of Interest

MD reports personal fees from Abingdon Health, outside the submitted work. HB, NP, and AAR are employees of M3 Global Research. The remaining authors declare that the research was conducted in the absence of any commercial or financial relationships that could be construed as a potential conflict of interest.
