# Supplementary material for: Health Care Professionals' Confidence and Preferences for Diagnostic Assays for SARS-CoV-2: A Global Study
Source: Front Public Health. 2021 Feb 26;9:569315. doi: 10.3389/fpubh.2021.569315 (PMC7952327; doi:10.3389/fpubh.2021.569315)
Supplement: Supplementary file 1 [file Data_Sheet_1.PDF]

Supplementary Methods

Table 1: Questions used in survey deployed to health care professionals

Question	Responses						
1. How confident do you personally feel in knowing which test should be undertaken during which stage of the illness	Completely confident	Fairly confident	Somewhat unsure	Completely unsure			
2. At what stage of the illness is each test most likely to be accurate<br><br>a. Antigen test<br>b. Antibody test	Before symptoms are present	During the early stages of symptoms	As the patient recovers from their illness	After the patient has recovered (> 1 month)	Not sure		
3. How important do you think each type of test is in controlling the COVID-19 pandemic	Critically important	Very important	Somewhat important	Not at all important			
4. All tests are subject to false positive and false negatives. For each type of test, please indicate you preference<br><br>a. Antigen test<br>b. Antibody test	Optimised for high specificity	Both specificity and sensitivity are equally important	Optimised for high sensitivity				
5. How likely are you to use each test in your clinical practice<br><br>a. Antigen test<br>b. Antibody test	Extremely likely	Very likely	Somewhat likely	Somewhat unlikely	Not at all likely		
6. For COVID-19 tests in general, would you prefer a home testing option or a laboratory test	A laboratory test	A home testing kit					
7. For COVID-19 tests in general, what type of test would you prefer/	A saliva test	A finger prick test	A blood sample test				
8. What would a positive test mean to/for an individual once they have recovered: rank from 1 (most important) to 7 (least important)<br><br>A. Antigen test<br>B. Antibody test	Confidence in interacting with with family and friends outside of work	Confidence in returning to work	Confidence in using public transport	Less concern with handwashing	Less concern with PPE	Less concern with social distancing	Other
