# Supplementary material for: Health Care Professionals' Confidence and Preferences for Diagnostic Assays for SARS-CoV-2: A Global Study
Source: Front Public Health. 2021 Feb 26;9:569315. doi: 10.3389/fpubh.2021.569315 (PMC7952327; doi:10.3389/fpubh.2021.569315)
Supplement: Supplementary file 2 [file Data_Sheet_2.PDF]

	n	% total
**Aesthetic Medicine**	62	0.36%
**Allergy/Immunology**	95	0.55%
**Anesthesiology**	398	2.32%
**Cardiology**	545	3.17%
**Dermatology**	231	1.34%
**Emergency Medicine**	400	2.33%
**Endocrinology/Diabetes**	293	1.70%
**Family/Primary Care/General Practice**	2220	12.92%
**Gastroenterology**	363	2.11%
**Hematology-Oncology**	395	2.30%
**Hepatology**	21	0.12%
**Hospitalist**	254	1.48%
**Infectious Diseases**	200	1.16%
**Internal/Critical Care**	585	3.40%
**Medical Genetics**	16	0.09%
**Nephrologist**	203	1.18%
**Neurology**	400	2.33%
**OBGYN**	486	2.83%
**Ophthalmology**	392	2.28%
**Orthopedics**	418	2.43%
**Otolaryngology/ENT**	216	1.26%
**Pain Management**	61	0.35%
**Pathology**	78	0.45%
**Pediatrics**	741	4.31%
**Physical Medicine & Rehabilitation**	95	0.55%
**Psychiatry**	421	2.45%
**Pulmonology**	252	1.47%
**Radiology**	198	1.15%
**Rheumatology**	153	0.89%
**Surgery**	484	2.82%
**Urology**	171	0.99%
**Vascular Medicine**	38	0.22%
**Other**	440	2.56%
**No response**	5861	34.10%
**Total**	17186	100.00%

Supplementary Table 1: Self-reported medical specialisms of study participants.
