# Supplementary figures and images for: Health Care Professionals' Confidence and Preferences for Diagnostic Assays for SARS-CoV-2: A Global Study
Source: Front Public Health. 2021 Feb 26;9:569315. doi: 10.3389/fpubh.2021.569315 (PMC7952327; doi:10.3389/fpubh.2021.569315)

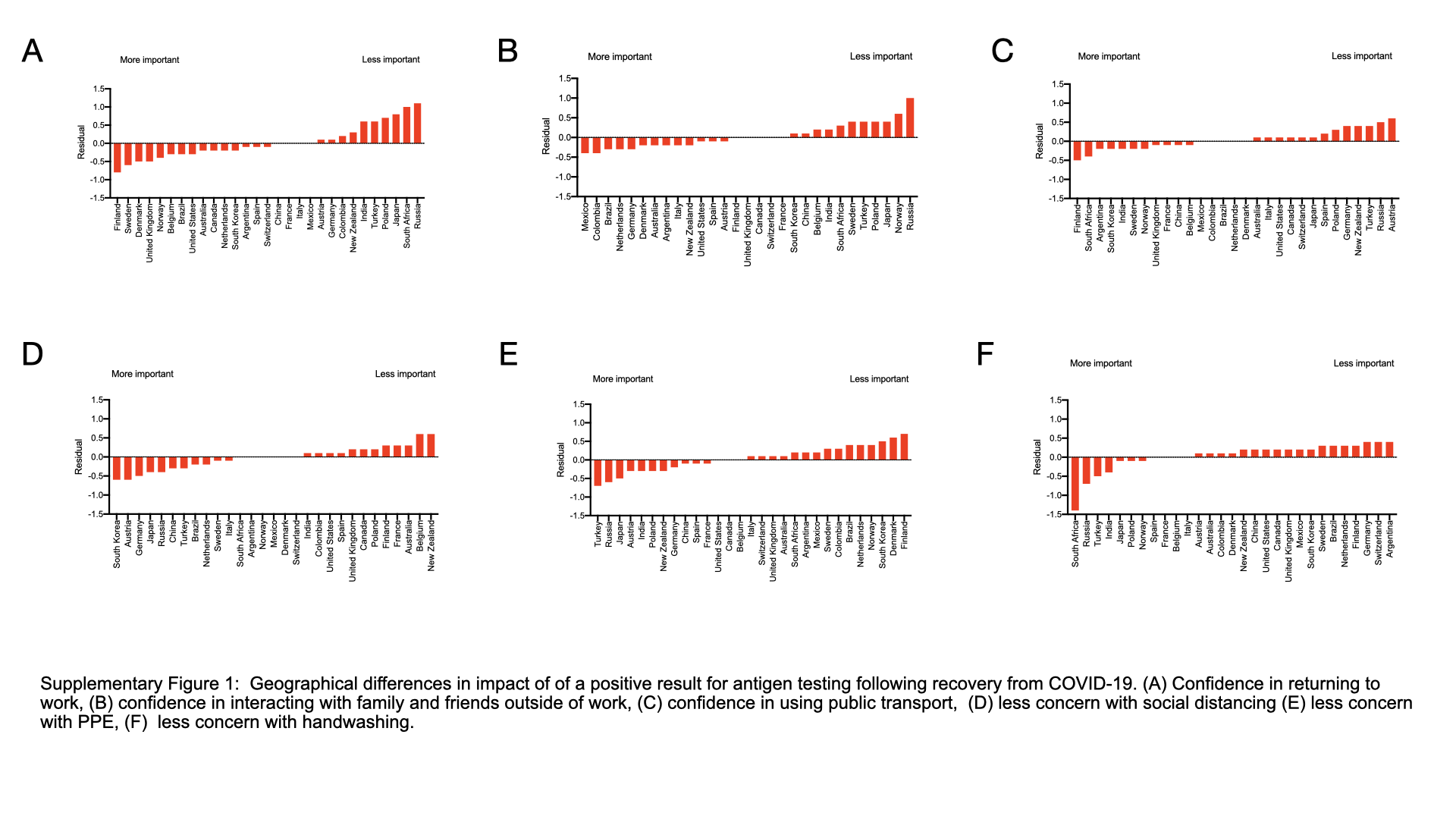

Supplement: Supplementary file 3 [file Image_1.TIFF]

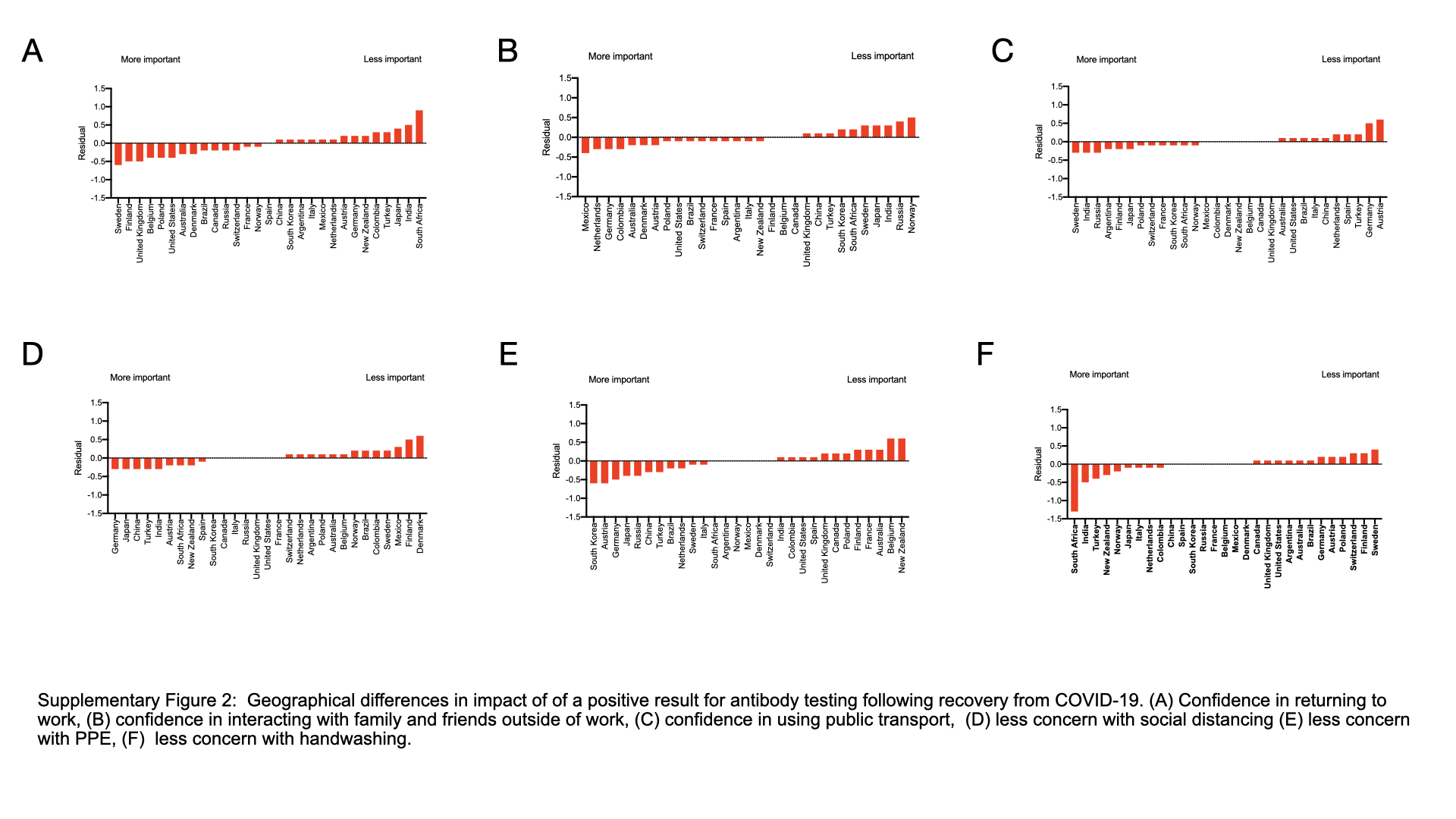

Supplement: Supplementary file 4 [file Image_2.TIFF]

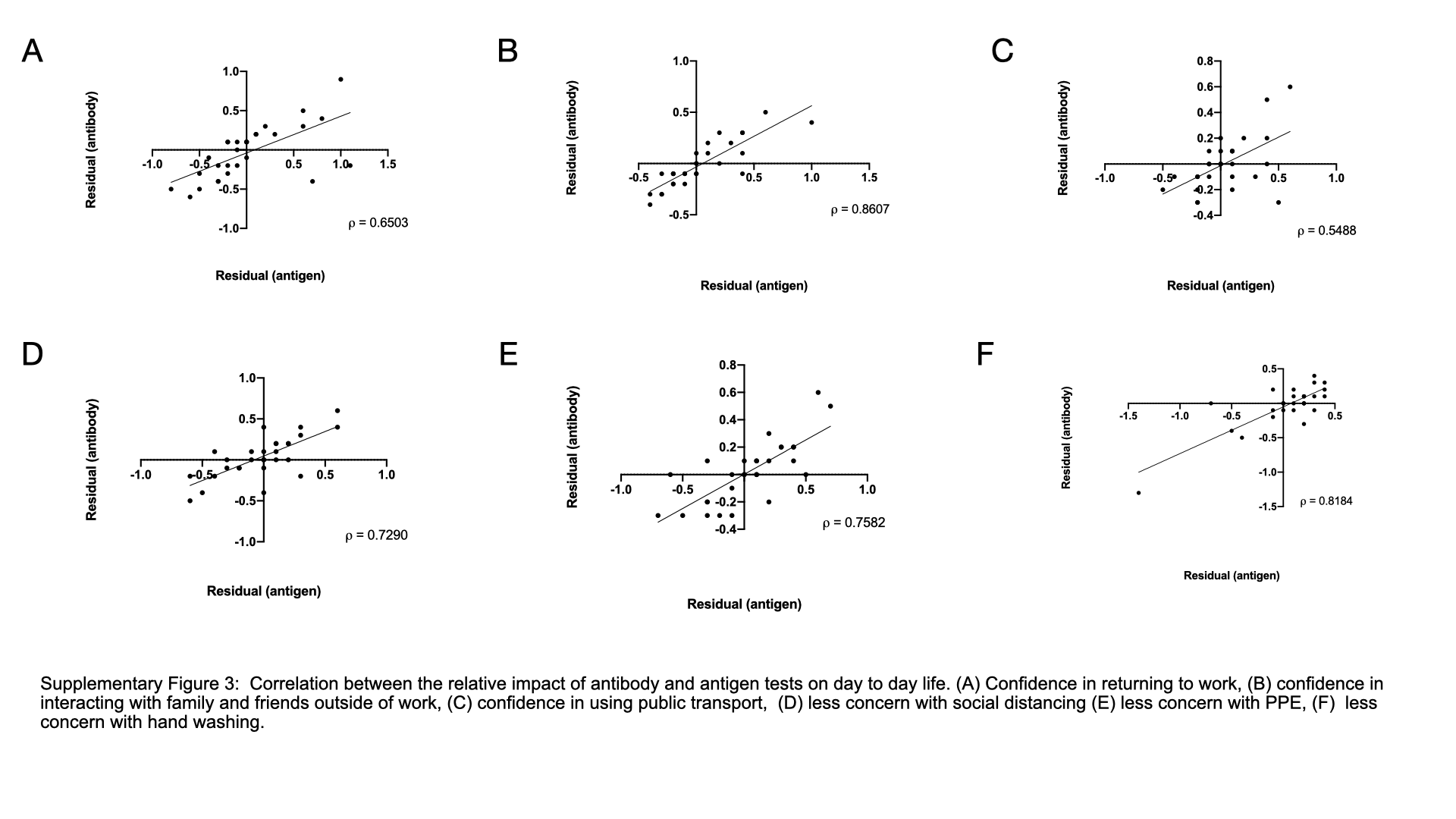

Supplement: Supplementary file 5 [file Image_3.TIFF]
